# VTBuilder: a tool for the assembly of multi isoform transcriptomes

**DOI:** 10.1186/s12859-014-0389-8

**Published:** 2014-12-03

**Authors:** John Archer, Gareth Whiteley, Nicholas R Casewell, Robert A Harrison, Simon C Wagstaff

**Affiliations:** Department of Parasitology, Liverpool School of Tropical Medicine, Pembroke Place, Liverpool, L3 5QA2 UK

**Keywords:** Transcriptomics, *de novo*, Contigs, Next generation sequencing, Software, Java, Chimeras, Haplotypes, Non-chimeric, Transcripts

## Abstract

**Background:**

Within many research areas, such as transcriptomics, the millions of short DNA fragments (reads) produced by current sequencing platforms need to be assembled into transcript sequences before they can be utilized. Despite recent advances in assembly software, creating such transcripts from read data harboring isoform variation remains challenging. This is because current approaches fail to identify all variants present or they create chimeric transcripts within which relationships between co-evolving sites and other evolutionary factors are disrupted. We present VTBuilder, a tool for constructing non-chimeric transcripts from read data that has been sequenced from sources containing isoform complexity.

**Results:**

We validated VTBuilder using reads simulated from 54 Sanger sequenced transcripts (SSTs) expressed in the venom gland of the saw scaled viper, *Echis ocellatus*. The SSTs were selected to represent genes from major co-expressed toxin groups known to harbor isoform variants. From the simulated reads, VTBuilder constructed 55 transcripts, 50 of which had a greater than 99% sequence similarity to 48 of the SSTs. In contrast, using the popular assembler tool Trinity (r2013-02-25), only 14 transcripts were constructed with a similar level of sequence identity to just 11 SSTs. Furthermore VTBuilder produced transcripts with a similar length distribution to the SSTs while those produced by Trinity were considerably shorter. To demonstrate that our approach can be scaled to real world data we assembled the venom gland transcriptome of the African puff adder *Bitis arietans* using paired-end reads sequenced on Illumina’s MiSeq platform. VTBuilder constructed 1481 transcripts from 5 million reads and, following annotation, all major toxin genes were recovered demonstrating reconstruction of complex underlying sequence and isoform diversity.

**Conclusion:**

Unlike other approaches, VTBuilder strives to maintain the relationships between co-evolving sites within the constructed transcripts, and thus increases transcript utility for a wide range of research areas ranging from transcriptomics to phylogenetics and including the monitoring of drug resistant parasite populations. Additionally, improving the quality of transcripts assembled from read data will have an impact on future studies that query these data. VTBuilder has been implemented in java and is available, under the GPL GPU V0.3 license, from http:// http://www.lstmed.ac.uk/vtbuilder.

**Electronic supplementary material:**

The online version of this article (doi:10.1186/s12859-014-0389-8) contains supplementary material, which is available to authorized users.

## Background

With the advent of new sequencing technologies that have parallelized the way in which sequencing chemistry is performed [1,2], attempts have been made to gain new insight into previously unstudied transcriptomes at both an inter- and intra- species level [3,4]. Prior to being utilized within transcriptomic studies however, the millions of short DNA fragments generated, termed reads, must to be assembled into longer contiguous sequences that are representative of the underlying transcripts present within the transcriptome. Despite recent advances in transcriptome assembly tools [5-9] and their application to a wide range of research areas including the characterization of diversity within viral populations, plants, mice and humans [10-16], accurately reconstructing transcript diversity within complex multi-isoform transcriptomes has remained a significant challenge [17-19]. Assembling snake venom gland transcriptomes, a complex multi-isoform toxin cocktail arising from ancestral gene duplication events and divergent evolution [20-23], exemplifies this challenge. Although linked by common ancestry, there is significant sequence variation within toxin families, such as the snake venom metalloproteinases (SVMP), C-type lectins, serine proteases (SP), phosoholipase A_2_s, bradykinin potentiating peptides and three-finger neurotoxins which often manifest in functionally distinct properties [24-29]. Sequence and functional diversity is complicated further at an inter- and intra-species level, as the expression of toxin isoforms is influenced by factors such as diet, habitat, sex, age and phylogeography [30-38]. Combined, this results in snake venom being complex [39-42] and it is this complexity that poses difficulties for current transcriptome assembly tools. Reconstructing accurate toxin transcripts is important because venom gland transcriptomes are a critical resource for the development of improved snakebite therapies [43,44].

Current assembly tools implement algorithms largely based around two different approaches [45,46]. In the first, reads are aligned to positions within reference transcripts to which they are most similar. This is termed mapping. This results in scaffold-like alignments from which networks representing sequence variation are constructed. Paths across these networks are used to construct transcripts that represent the diversity present within the transcriptome. However, for many transcriptomes including snake venom gland transcriptomes, complete reference datasets rarely exist. When they do, being derived from low coverage Sanger/EST studies [42,47-49], they may not have captured the full extent of variation within the transcriptome being studied. In this case, new transcripts cannot be discovered using a reference based approach as reads with insufficient similarity to sequences within the reference dataset will be discarded. Conversely, reads that are less divergent from transcripts within the reference dataset are more likely to map [50]. Thus, the extent of divergence between venom gland transcriptomes even at an intra-species level [3,39,42], will result in a biased loss of read data during mapping. This in turn, will result in a decreased accuracy in the estimation of transcript expression; even when mapping to a transcriptome from the nearest available species as a pseudo-reference dataset.

To resolve problems associated with the lack of a suitable reference transcriptome *de novo* based assembly can be applied. This usually involves the construction of de Bruijn networks that represent clusters of diversity, e.g. individual protein families within the data [17]. On these networks nodes represent short sequence fragments, called k-mers, which are derived from reads, while edges represent shared identity between k-mers. These networks encompass all of the diversity present with the read data and traversals are used to construct transcripts. However, in the presence of isoform variation, maintaining non-chimeric paths across the subsequently complex networks becomes difficult [17,51]. This is because a rise in diversity increases the number of nodes, which increases the combinatorials involved in path traversal. Distinguishing chimeric from non-chimeric paths is difficult as chimeras are in effect artificial recombinants generated between the true isoforms and, despite having superficial resemblance to true isoforms, relationships between co-evolving sites, functional motifs and other evolutionary factors are not maintained. This is due of the introduction of breakpoints within chimeras that are solely an artefact of the assembly process and not as a result of transcriptome evolution. Thus, resolving the true evolutionary relationship between transcripts becomes difficult. Long k-mers are often used to aid this task [5,52], but success is not guaranteed [17,51].

**Figure 1:**
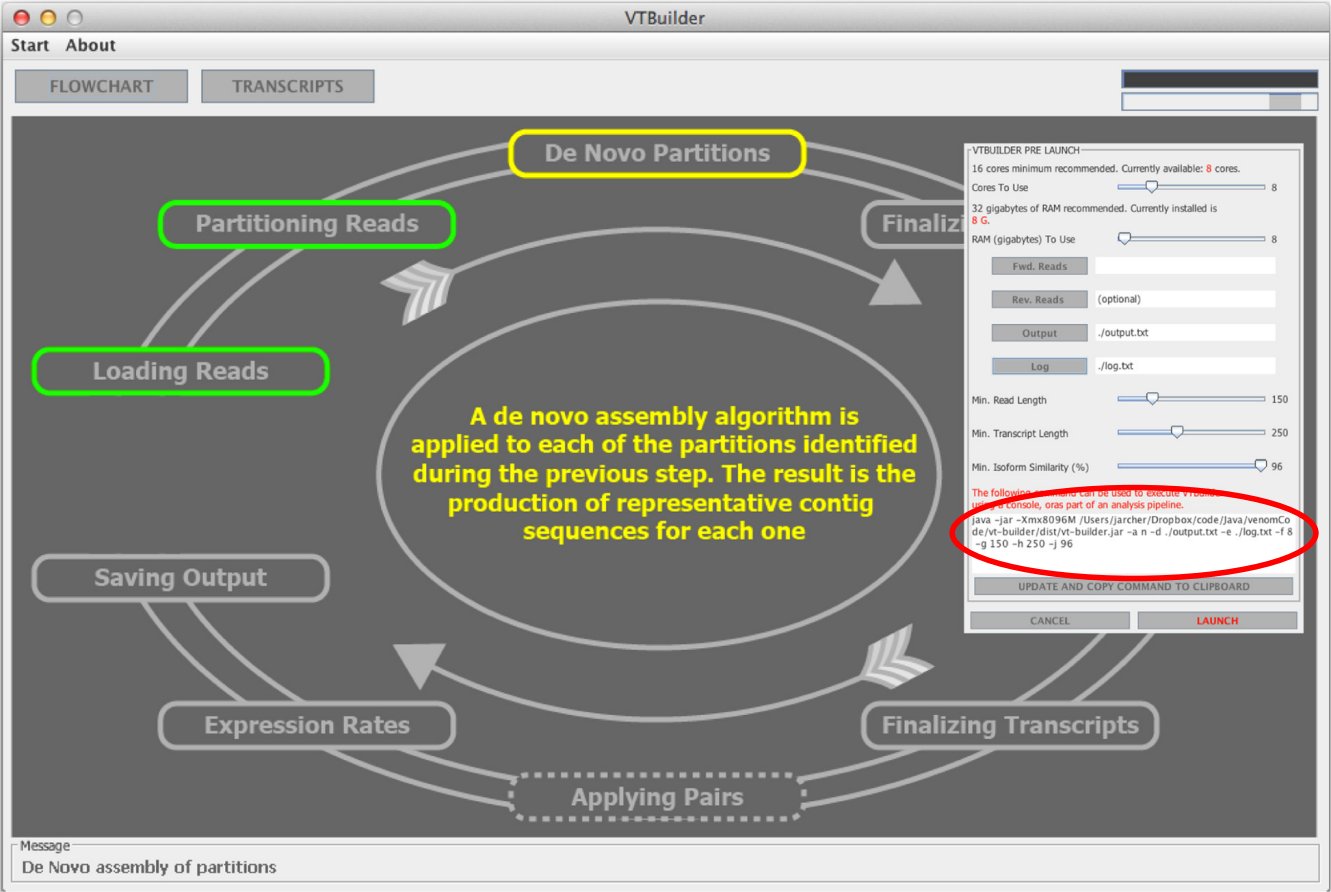
**VTBuilders Graphical User Interface (GUI).** Green boxes indicate completed steps of the pipeline while grey indicate those yet to be performed. The yellow box shows the step that is currently running while the yellow text provides a brief description of the step currently running. The inset panel displays the setup area that the user is presented with when they initially double click the jar file. The red circle indicates the command that is required if the user wishes to use the software without a GUI via the command line.

To address the issues associated with current assembly tools we designed VTBuilder (Figure 1), a user-friendly software for the assembly of non-chimeric transcripts. No reference transcriptome is required and the input can be single or paired end read data in FASTQ format. The software can be launched by executing a single jar file at which point the user will be presented with a Graphical User Interface (GUI) (Figure 1: inset) from which the user can interact with the software via the GUI or using the dynamically generated command in a terminal window (Figure 1: inset, red circle). Installing and running VTBuilder is described in a user guide that is available on the project website. VTBuilder implements a six step bioinformatics pipeline that is described in detail within the implementation section. Briefly, *(i)* Reads are partitioned into broad groups of shared diversity such as protein families. *(ii) De novo* assembly on each partition is performed to produce a set of guide sequences. *(iii)* A set of scaffold-like alignments, similar to those used in reference based assembly [45,46], is produced by mapping each read to the guide sequence that it is most similar to; *(iv)* For each scaffold like alignment a network is created that represents the isoform diversity present; *(v)* Transcripts are constructed by traversing these networks; and (vi) Transcript expression is calculated by remapping the read data to the constructed transcripts and then counting the reads mapped to each followed by length normalization.

Producing non-chimeric transcripts is essential if the resolving power of next generation sequence (NGS) data is to be used to dissect the evolutionary dynamics within complex transcriptomes with no available reference. At the time of writing we are unaware of any freely, or otherwise, available software that makes this possible. We benchmark the accuracy of our software, against a current popular *de novo* assembler, Trinity [5,52], which implements a method to traverse multiple de Bruijn graphs. In our analysis we used read data simulated from 54 known venom gland Sanger sequenced transcripts (SSTs) representing isoforms of the most frequent and diverse viper venom gland protein families. Using VTBuilder, over 90% of SSTs were accurately reconstructed from the simulated reads into transcripts sharing 99% or greater sequence similarity with one of the known SSTs, compared with only 25% recovered using Trinity. Following this, we benchmarked accuracy and performance of VTBuilder by constructing transcripts from 2.5 million paired end Illumina MiSeq reads sequenced from the venom gland of the African puff adder, *Bitis arietans*. This is the first assembly of an NGS-derived snake venom gland transcriptome using a new tool to overcome the inclusion of chimeric transcripts that typically confound the interpretation of multi-isoform venom gland transcriptomes. The correct assembly of transcripts is an important step towards the realization of the full potential that NGS technology has to offer in resolving the biological complexity of highly variable transcriptomes.

## Implementation

### Overview

**Figure 2:**
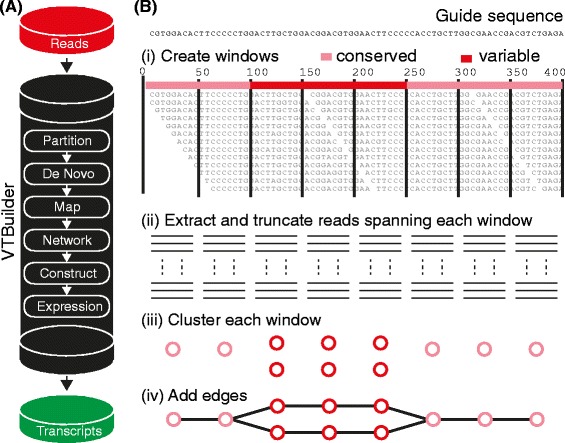
**Implementation. (A)** Schematic diagram of the VTBuilder assembly pipeline. **(B)** For each scaffold-like alignment produced during mapping a network is constructed. *(i)* Non-overlapping windows are positioned along the assembly. *(ii)* Reads spanning each window are extracted and truncated. *(iii)* These are then clustered to produce nodes. *(iv)* Edges are placed between clusters that share reads.

The overall aim is to broadly capture transcript diversity by building a set of guide sequences from the read data and then to use these guides as a template to assist in the more accurate assembly of transcripts in a manner similar to reference based assembly [45,46]. To achieve this, our software implements six steps schematically represented in Figure 2A.

(i.) *Partitioning:* This involves grouping reads into partitions (or clusters) that broadly reflect the protein diversity present within the data (i.e. at the protein family level not individual isoforms). Partitioning is done using an approach that is similar to seed based clustering [53]. In the latter a read is randomly selected to seed (start) a partition and subsequent read inclusion to the partition is dependent on similarity to this read. We modified the approach to include reads derived from different positions on the same underlying transcript and thus share little sequence similarity. In VTBuilder a partition is initiated by randomly selecting a read from the input forward reads. This read is termed the partition seed. All remaining forward reads are searched and added to the partition, and removed from the input set, if they share a region of similarity (70% identity across 100 bases) to this seed. The 100 base window size allows for positional flexibility between the reads and the seed, i.e. reads are allowed to match to either side of the seed. Once complete, up to 12 reads are randomly selected from the reads just added, and a new search of the unpartitioned reads is initiated against these. Selecting reads added in the previous cycle reduces redundancy in the search. As cycles continue, the partition is expanded until no new reads can be added. At this point a new read is randomly selected from unallocated reads and used to seed a new partition and the process repeats. The partitioning step finishes when there are no unallocated reads left. Partitioning results in groups of unassembled reads that are directly related by transcript or indirectly related by protein family. Partitions containing 3 or more reads progress to step 2. Reads within smaller partitions are not used within step 2 but are reintroduced during step 3 (mapping) along with all other input data. Thus, no reads are permanently discarded from the pipeline at this point.
(ii.) *De novo:* Here guide sequences, which will be used as templates for subsequent mapping, are constructed from partitions. This is done using a greedy overlap method of assembly. Within each partition a read is randomly selected to initiate guide construction. We call this read the growing guide sequence (GGS). The remaining reads within the partition are searched against the GGS. If a read with high similarity to the GGS is identified (98% or greater similarity across a 100 nucleotide window) it is joined to the GGS, using the region of similarity as an anchor point. If this results in an extension to the GGS then the read is removed from the partition and the joined sequence replaces the current GGS. A new search against all the remaining reads in the partition is then initiated. This process iterates until a search against all remaining reads does not result in an extension to the GGS. If the partition still contains reads, a new guide is then initiated by selecting a random read and the process repeats. Thus, a single partition can result in more than one guide sequence. Once guide sequences have been created from all partitions a final *de novo* step is performed in order to join any partial guide sequences.
(iii.) *Mapping:* During mapping all input reads are aligned against the guide sequence that they are most similar, to at positions that minimize diversity. In VTBuilder, we used a mapping algorithm that we previously developed to map read data containing high amounts of variation. The algorithm uses short fragments (10 bases in length), termed k-mers, extracted from each guide sequence to form a library containing k-mer positional information for each guide. This library is then compared with k-mers derived from individual reads in order to find the most probable location for each read on the guide sequence to which it is most closely related. This standard k-mer indexing approach is described in detail in [50,54]. This results in alignment-like structures, termed scaffolds, where reads are positionally correct to each other and to the guide sequence to which they are most related (Figure 2B, i).
  The next two steps are designed to minimize chimeric transcripts by retaining as much diversity and positional information as possible within networks (step iv) whilst ensuring only the most robust (non-chimeric) paths are traversed to become transcripts (step v).
(iv.) *Networking:* Here we represent each scaffold-like alignment produced in (iii) as a graph-based structure by transforming isoform-specific differences (diversity) in alignments into nodes and edges, that are subsequently traversed and assembled into finished transcripts in step (v). Non-overlapping neighbouring windows of pre-defined size (see below) are first defined across the scaffold-like alignment (Figure 2B, i) after which reads spanning each window are extracted, truncated (Figure 2B**,** ii) and clustered using hamming distance (Figure 2B**,** iii). Clusters are represented as nodes on the network. Window size is calculated as one third of the minimum read length (user defined) thus ensuring that any three neighboring windows, and their subsequent nodes, have the potential to contain different regions of the same physical read. This information is used during network traversal in step *(v)*. Edges are placed based on this physical linkage, where any two connected nodes physically share at least two reads, albeit different regions on these reads (Figure 2B, iv). Within the software, the minimum read length is limited to 120 bases to ensure there is sufficient sequence information within each window to cluster based on diversity. This step constructs multiple networks where nodes represent regions of diversity in reads mapped to the guide sequences.
(v.) *Constructing Transcripts:* A final list of assembled transcripts is outputted by traversing the networks created in *(iv)*. Importantly for the reduction of chimeric transcripts, the physical linkage of reads between adjacent node triplets guides traversals i.e., with the exception of the first two nodes added to a path, a node will only be added if it contains read fragments that are physically linked to fragments present within the two previously added nodes constructed in (iv) from 3 neighboring windows. This ensures that each individual path is a traversal through nodes containing reads derived from a single isoform within the underlying data, and is the key step in limiting chimeric paths. Paths are initiated for each cluster of diversity present within the first window. For each path initiated, the addition of a second node is dependent on reads overlapping with the first. If paired end reads are available they are used to confirm paths. For each read on a path an attempt is made to map its pair. If less than 30% of the pairs map then the path is discarded.
(vi.) *Expression:* Calculation of relative transcript expression is achieved by remapping all the input reads to the finished transcripts. The expression level for a single transcript is taken as the number of reads mapping to that transcript normalized by the length of the transcript. These are outputted on the transcript titles as a percent relative to all other transcripts.

## Results and discussion

### Case study 1: simulated transcriptome assembly

To demonstrate the ability of VTBuilder to construct transcripts from reads derived from a diverse range of protein families, including those harboring extensive isoform variation, we devised a controlled study using 54 known full-length Sanger sequenced transcripts (SSTs) expressed within the venom gland transcriptome of the West African saw-scaled viper *Echis ocellatus* [42,49,55]. These sequences were selected to represent the most commonly observed proteins within snake venom [3] and comprise different families, length distributions and isoform diversity (Table 1). They include genes from the major expressed toxin groups known to harbor isoform variation, such as SVMPs and SPs, as well as conserved single copy genes not thought to be involved in predation or defense, such as Poly A Binding Protein and Protein Disulfide Isomerase. Where isoform variants existed within a group (e.g. there are 10 P-III class SVMPs in the dataset), the diversity present was visualized by creating alignments and neighbor joining trees using ClustalX [56] (Additional file 1: Figure S1). Using read data simulated from these 54 known transcripts as the input, we assessed the accuracy at which VTBuilder (V0.1.8.4), as well as Trinity (Release: r2013-02-25) [5,52], was able to reconstruct transcripts by directly comparing the results back to the known SSTs.

**Table 1:** **The 54 known SSTs used to seed the simulation of reads as described in case study 1**

**Protein**	**No. of isoforms**	**Length range**
SVMP I	1	1600
SVMP II	3	1600 - 2000
SVMP III	10	1600 - 2300
Serine Protease	9	700 - 1400
Phospholipase A2	3	600
CTL	16	500 - 700
NGF	1	700
CRISP	1	850
VEGF	1	650
LAAO	1	1450
Creatine Kinase	1	790
β-Actin	1	630
HSP90 Endoplasmin	1	780
ATPase6	1	720
Cytochrome C Oxidase	1	880
Poly A Binding Protein	1	680
Cytochrome B	1	800
Protein Disulfide Isomerase	1	1650

Column 2 contains the number of sequences representing each protein family. Column 3 displays the lengths of the sequences included.

**Figure 3:**
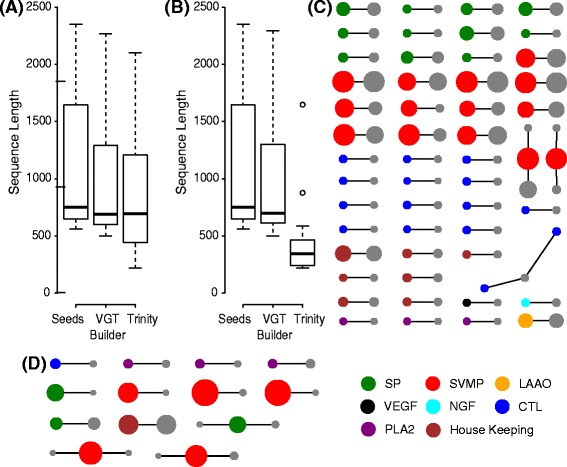
**Transcript reconstruction on simulated reads. (A)** Lengths of all transcripts constructed by VTBuilder and Trinity compared to those of the SSTs. The top and bottom of the boxes represent the 25th and 75th percentiles respectively, while the top and bottom whiskers represent the third quartile +1.5 times the inter quartile range (IQR) and the first quartile - 1.5 times the IQR respectively. Outliers beyond these points are represented as black circles. **(B)** Lengths of transcripts constructed by VTBuilder and Trinity that had a sequence similarity of 90% or greater to the SSTs. **(C)** Network showing the relationship between the VTBuilder transcripts and the SSTs. Grey nodes represent the VTBuilder transcripts. Colored nodes represent the protein families to which the individual SSTs belong (see key). Node size is proportional to sequence length. Edges represent a 90% or greater sequence similarity. **(D)** Same as **(C)** but using Trinity to construct the transcripts.

In brief, 50,000 reads of length 250 bases were copied from the 54 SSTs at random locations. For each read, its pair was copied randomly from a window 500 bases wide anchored on the last base of the read itself. Read coverage across each SST was normalised by length resulting in an upper bound of 1930 reads covering the longest SST and a lower bound of 480 covering the shortest. This is equivalent to an upper per site coverage of 209 and a lower per site cover of 190, typical of the coverage observed in an NGS dataset. Note 50,000 reads is far less than would be expected within an NGS dataset but here the reads are covering far fewer transcripts (54 SSTs) than the thousands of transcripts typically found within a transcriptome. This read/transcript ratio was selected to represent approximately 7 M reads covering a transcriptome of around 7500 genes. VTBuilder, running default parameters (min. read ln. 150; min. transcript ln. 250; min isoform sim. 96%) and on a desktop with 16 cores, 32 gigabytes of RAM and Biolinux 7 (Ubuntu 12.04) [57], was then used to construct transcripts from the simulated paired end reads (see user guide). VTBuilder constructed 55 transcripts of comparable length distribution (ranging from 500 to 2298 bp) to the input SSTs (Figure 3A). Using the same simulated paired end data as input, Trinity (using default parameters) resulted in the construction of many more (112) transcripts that ranged in length from 217 to 2104 bp (Figure 3A).

Next, we assessed the accuracy of transcript reconstruction by evaluating the level of sequence similarity between the 54 original SSTs and the transcripts constructed by both VTBuilder and Trinity. 50 of the 55 transcripts constructed by VTBuilder matched 48 of the SSTs with a similarity of 99% or greater. 53 transcripts matched 51 SSTs with a similarity of 95% or greater while 54 transcripts matched 53 of the SSTs with a similarity of 90% or greater. In comparison only 14 of the 112 transcripts constructed by Trinity matched 11 of the SSTs with a similarity of 99% or greater. 16 transcripts matched 13 SSTs with a similarity of 95% or greater. This remained unchanged at 90% similarity. Of the transcripts assembled from both software that matched the SSTs with a similarity of 90% or more, the length distributions of those produced by VTBuilder were more similar to the SSTs than those produced by Trinity (Figure 3B). These similarity and length distributions suggest that VTBuilder produces longer and more accurate transcripts than Trinity when run on the simulated reads and a thus a more comprehensive and accurate reconstruction of the original SSTs.

To further refine our understanding of the multi-isoform assembly process, we investigated whether reconstructed transcripts for both Trinity and VTBuilder displayed a one-to-one sequence similarity relationship with the original SSTs or whether chimeric assemblies producing many-to-many relationships existed. Within individual protein families containing multiple isoform variants, a many-to-many relationship would indicate a failure to distinguish between different isoforms. Reconstructed transcripts and original SSTs were used as nodes on a network where edges represent a sequence similarity of 90% or more. When the 54 transcripts (grey) constructed by VTBuilder were placed on a network along with the 53 SSTs that they matched (colors, see key), they largely displayed a one-to-one relationship (Figure 3C). Node size is proportional to sequence length further demonstrating that VTBuilder was capable of reconstructing transcripts of virtually identical composition and length as each original SSTs in comparison to Trinity where shorter, nearly exact matches of local similarity were more typical of the dataset (Figure 3D).

To investigate the effects of sequence error on VTBuilder performance we repeated our analysis using the same 54 SSTs but with a per site error rate introduced within each simulated dataset. At the per site mismatch error rates of around 0.2% typical of Illumina technology [54], VTBuilder constructed a total of 53 transcripts, 50 of which retained a greater than 90% similarity to the SST sequences with typically one-to-one relationships (Additional file 2: Figure S2). This level of accuracy in transcript reconstruction was maintained up to a high per site error rate of 1%, beyond which the total number of transcripts constructed increases as does the discrepancy between the number of VTBuilder transcripts sharing a 90% similarity with the SSTs. Introducing higher levels of artificial variation into the population, such as a 2% per site error rate, will result in at least 99.35% of the reads containing on average 5 errors across the 250 bases [58]. This level of diversity is sufficient for VTBuilder to recognise transcripts as separate isoforms and leads to the sudden and expected rise in transcripts and drop in accordance with SSTs (Additional file 2: Figure S2).

Taken together, the results of our simulations indicate that VTBuilder can reconstruct transcripts that are highly similar both in length and sequence composition to the 54 input SSTs. The software can also accurately reconstruct transcripts when faced with a higher than expected degree of sequencing error.

### Case study 2: assembly of a snake venom gland transcriptome from NGS data

**Figure 4:**
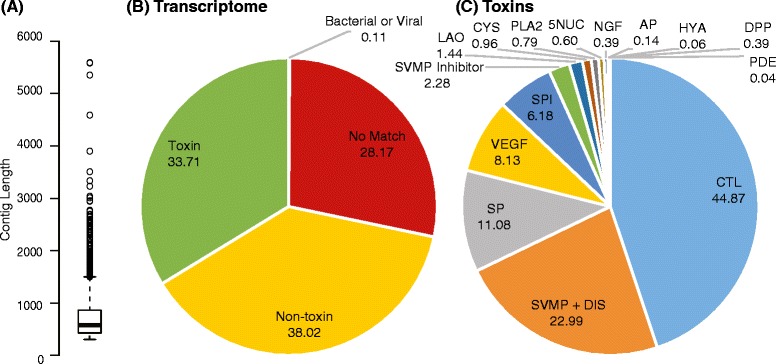
**Scaling up to real data.** Reads from the venom gland of *Bitis arietans* were assembled using VTBuilder and annotated using BLAST2GO [59]. **(A)** Box and whisker plot depicting the length distribution of the constructed transcripts (see Figure 3 for details of whiskers). **(B)** Transcripts were categorized into four groups; (i) Toxins, (ii) Non-Toxins, (iii) No significant match, and (iv) Bacterial or Viral DNA. **(C)** The Toxin group in **(A)** was split into sub categories representing the different protein families present.

To demonstrate the application of our software to real world data, we sequenced the venom gland transcriptome of the Nigerian puff adder *Bitis arietans*. Venom glands were dissected and homogenised, total RNA extracted (TRIzol Plus RNA purification kit; Invitrogen), DNase treated (PureLink DNase Set; Invitrogen), and poly(A) selected (Dynabeads mRNA DIRECT purification kit; Life Technologies). Sequencing was performed on the Illumina MiSeq platform with 250 bp paired-end reads producing 7,114,760 reads in total (Centre for Genomics Research, University of Liverpool). These were processed to remove low quality and unpaired reads leaving a total of 3,511,257 pairs. Post quality filtering resulted in a mean read length of 150 nucleotides. Reads were loaded into both VTBuilder and Trinity for assembly. VTBuilder constructed 1481 transcripts ranging in length from 300 to 5,598 nucleotides (mean length: 751) while Trinity constructed 61,709 transcripts ranging in length from 201 to 8815 nucleotides (mean length: 440) (Additional file 3: Figure S3 and Figure 3A), 31,477 of which were less than 300 nucleotides in length. Transcripts produced by VTBuilder were annotated using BLAST2GO [59] (BlastX; RefSeq Database Release 62, E-value <10×10^−5^) and subsequently sorted into four categories (Figure 4B): *(i)* toxins: i.e. transcripts homologous to transcripts found in the NCBI database coding for proteins previously identified as toxins. These made up 33.71% of the transcriptome and were comprised of 101 unique transcripts. Note: SVMP and SP inhibitors have been included within this group. *(ii)* non-toxins: i.e. transcripts homologous to proteins with no known pathology e.g. housekeeping genes. These made up 38.02% of the transcriptome and were comprised of 913 unique transcripts. *(iii)* no significant match found: i.e. transcripts with no match in the database or where the E-value of the match is >10×10^−05^. These made up 28.17% of the transcriptome and were comprised of 463 unique transcripts and *(iv)* bacterial or viral DNA: these made up 0.11% of the transcriptome and were comprised of 4 unique transcripts. Transcripts defined as toxins were subdivided into protein families (Figure 4C). All major viperid toxin families were accounted for, demonstrating that VTBuilder had accurately reconstructed the underlying transcriptome. Of note is the 101 unique toxin transcripts that contribute to just 6.81% of the total diversity present within the transcriptome (i.e. 101 out of 1481 unique transcripts), but make up 33.71% of the expressed transcriptome. These unique toxin transcripts fall largely into four main toxin families (Table 2), and highlight the importance of distinguishing between isoforms within the underlying data. For example 31 closely related but unique CTL isoforms were identified making up 44.87% of the toxins category. Our software demonstrates how NGS data can be exploited to provide a more accurate, high-resolution picture of complex transcriptomes, such as snake venom gland transcriptomes.

**Table 2:** **The 101 unique toxin transcripts recovered by VTBuilder from reads sequenced from the venom gland of** ***Bitis arietans*** **(column 1) and the overall percentage of the toxin DNA that they make up within the transcriptome**

**Toxin type**	**% of toxin transcripts**	**# of unique transcripts**
CTL	44.87	31
SVMP + DIS	22.99	26
SP	11.08	14
VEGF	8.13	5
SPI	6.18	9
SVMP Inhibitor	2.28	1
LAO	1.44	3
CYS	0.96	1
PLA2	0.79	3
5NUC	0.60	1
NGF	0.39	2
AP	0.14	1
HYA	0.06	1
DPP	0.06	2
PDE	0.04	1

Combined these made up 33.71% of the expressed transcriptome (Figure 4A) but only make up 6.81% of the total number of unique sequences present.

## Conclusion

We have demonstrated that transcripts constructed using VTBuilder accurately represent the variation present within venom gland transcriptomes. Unlike other approaches, our algorithm strives to maintain the relationships between factors such as to co-evolving sites and recombinant breakpoints within the underlying transcripts. VTBuilder has the potential to increase the usability of transcript sequences generated from read data across a wide range of research areas including; the detection of drug resistant variants within viruses and other disease causing parasites, where co-evolving sites confers resistance to particular classes of drugs [60-62]; the monitoring of disease progression, where variation across a range of sites can be indicative of progression and pathological outcome [50,63-68]; plant biology, where it has proven difficult to reconstruct full length transcripts representing complex transcript populations derived from genomes where polyploidy is present [69,70]; and reconstructing accurate evolutionary relationships on phylogenetic trees, and in detecting recombinant breakpoints, where the usage of long non-chimeric transcripts is essential. We have made the source code for VTBuilder available from https://code.google.com/p/vt-builder/ where researchers from a wide range of backgrounds can access and develop it for their own requirements. Finally, we consider VTBuilder as an important progression towards the full utilization of the potential that NGS data offers. This is because highlighting the problem of chimeric sequence assembly, as well as having a proposed solution, will begin to reduce the number of such sequences being deposited within public data repositories which will have a positive impact on future studies querying such sources.

## Availability and requirements

***Executable jar file and user guide is available from:***http://www.lstmed.ac.uk/vtbuilder

***Googlecode home page (source code)****:*https://code.google.com/p/vt-builder/

***Operating system(s)****:* Platform independent

***Programming language****:* Java

***Other requirements****:*

A Java runtime environment must be is installed. This is available from the Oracle website at: http://www.oracle.com/technetwork/java/javase/downloads/java-se-jre-7-download-432155.html. The tool is designed to run on a high spec desktop. We developed and tested it on a single processor Intel Xeon E2687W workstation equipped with 32GB of RAM, 16 cores and running Biolinux 7 [57]. We have tested both real world and simulated data on Biolinux 7 (Ubuntu 12.04) running Open JDK IcedTea v1.13.4, where the real world data described in case study 2 took just over 4 h to assemble, and simulated datasets on Biolinux 8 (Ubuntu 14.04) running Open JDK IcedTea v2.5.1. We have also tested simulated datasets on on Mac OS × 10.7.5 running java 1.7.0_09.

***License****:* GPL GPU V0.3.

### Availability of supporting data

Simulated read data used in Case Study 1 along with the corresponding 54 seed sequences (Table 1) are available at: http://www.lstmed.ac.uk/vtbuilder. The *Bitis arietans* read data presented in Case Study 2 is available on request from the authors.

## Additional files

Additional file 1: Figure S1. Sequence diversity within the 54 SSTs used in case study 1. Neighbour joining trees depicting sequence diversity present within the protein families that the SSTs represent. The scale bar represents nucleotide substitutions per site. Additional file 2: Figure S2. The effects of read error on transcripts generated by VTBuilder. The plot shows the total number of transcripts constructed by VTBuilder (black line) using simulated reads containing varying degrees of per site sequencing error (x-axis). The dashed line displays the number of transcripts with a greater than 90% similarity to an SST. Networks display the relationship between the SST’s and the transcripts in a similar manner to those depicted in Figure 3. Additional file 3: Figure S3. Summary of transcripts assembled using VTBuilder and those assembled using Trinity. (Whiskers have been defined in the legend of Figure 3).
