# Supplementary figures and images for: VTBuilder: a tool for the assembly of multi isoform transcriptomes
Source: BMC Bioinformatics. 2014 Dec 3;15(1):389. doi: 10.1186/s12859-014-0389-8 (PMC4260244; doi:10.1186/s12859-014-0389-8)

SVMP III

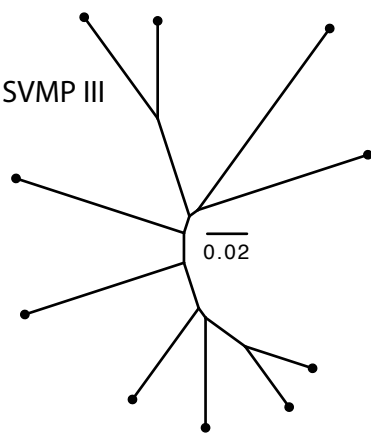

SP

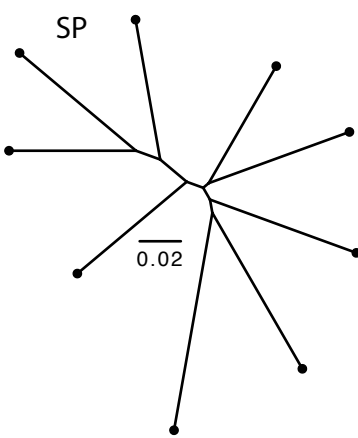

CTL

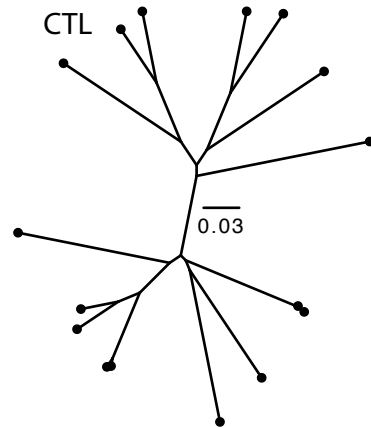

PLA2

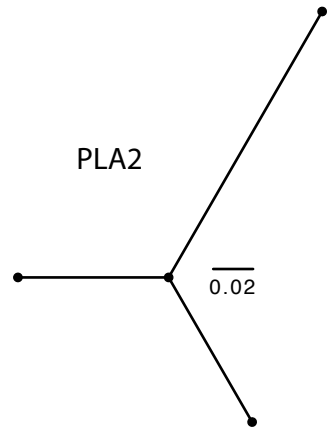

SVMP II

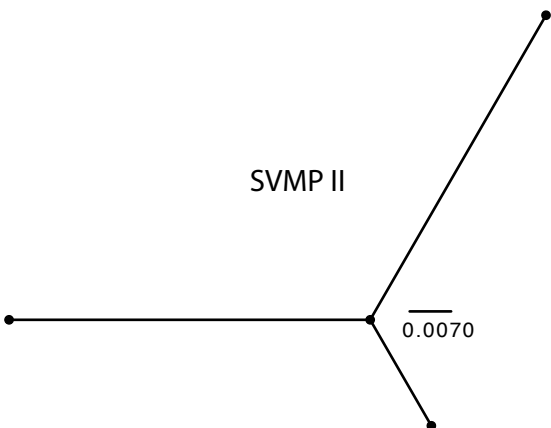

Supplement: Additional file 1: Figure S1. — Sequence diversity within the 54 SSTs used in case study 1. Neighbour joining trees depicting sequence diversity present within the protein families that the SSTs represent. The scale bar represents nucleotide substitutions per site. [file 12859_2014_389_MOESM1_ESM.pdf]

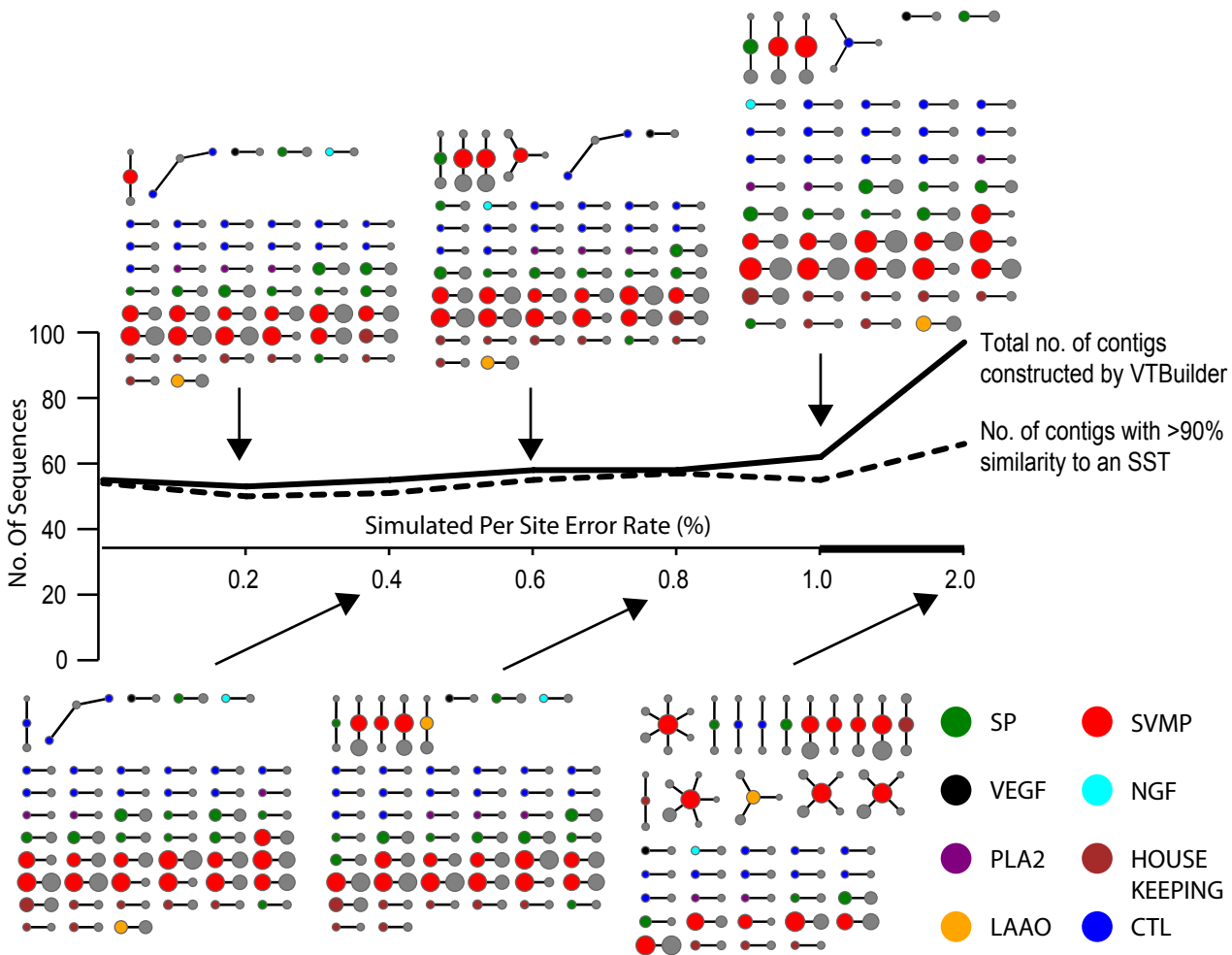

Supplement: Additional file 2: Figure S2. — The effects of read error on transcripts generated by VTBuilder. The plot shows the total number of transcripts constructed by VTBuilder (black line) using simulated reads containing varying degrees of per site sequencing error (x-axis). The dashed line displays the number of transcripts with a greater than 90% similarity to an SST. Networks display the relationship between the SST’s and the transcripts in a similar manner to those depicted in Figure 3. [file 12859_2014_389_MOESM2_ESM.pdf]

|                     | Trinity     | VTBuilder   |
|---------------------|-------------|-------------|
| <b>Total No.</b>    | 61709       | 1481        |
| <b>Mean Len.</b>    | 440         | 751         |
| <b>Median Len.</b>  | 297         | 580         |
| <b>Range (Len.)</b> | 201 to 8815 | 300 to 5598 |

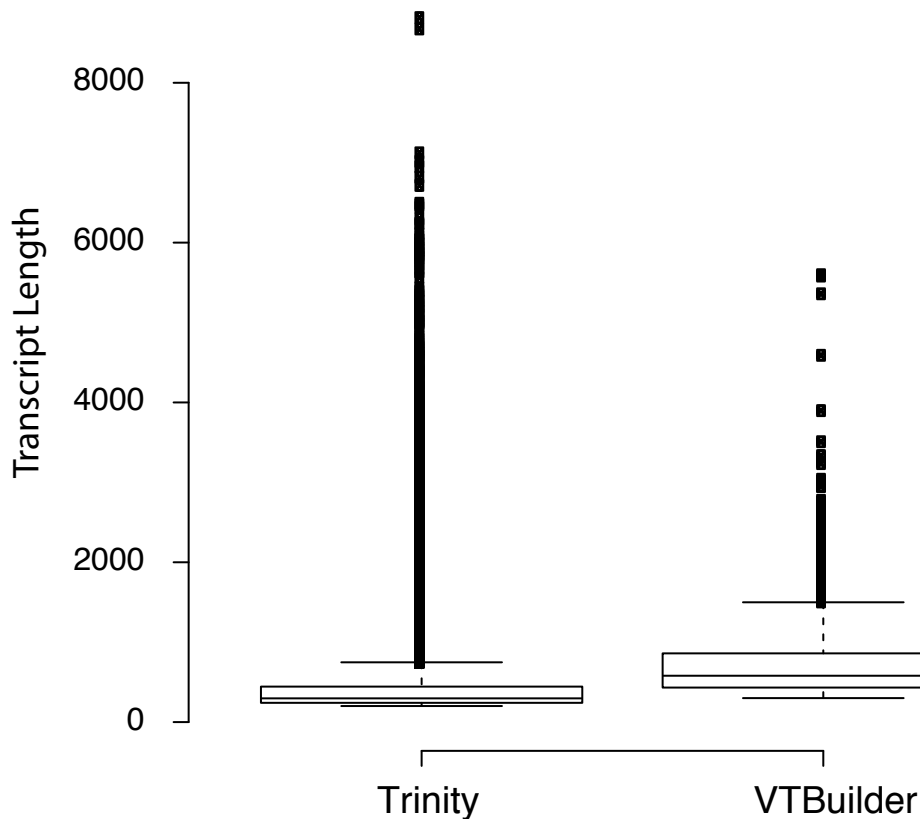

Supplement: Additional file 3: Figure S3. — Summary of transcripts assembled using VTBuilder and those assembled using Trinity. (Whiskers have been defined in the legend of Figure 3). [file 12859_2014_389_MOESM3_ESM.pdf]
